# Efficacy of Virtual Reality Rehabilitation after Spinal Cord Injury: A Systematic Review

**DOI:** 10.1155/2019/7106951

**Published:** 2019-11-13

**Authors:** Amanda Vitória Lacerda de Araújo, Jaqueline Freitas de Oliveira Neiva, Carlos Bandeira de Mello Monteiro, Fernando Henrique Magalhães

**Affiliations:** ^1^University of São Paulo, School of Arts, Sciences and Humanities–EACH–USP, São Paulo, Brazil; ^2^University of São Paulo, School of Physical Education and Sport–EEFE–USP, São Paulo, Brazil; ^3^University of São Paulo, Faculty of Medicine, Post-graduate Program in Rehabilitation Sciences–FMUSP–USP, São Paulo, Brazil

## Abstract

**Background:**

Spinal cord injury (SCI) is often associated with long-term impairments related to functional limitations in the sensorimotor system. The use of virtual reality (VR) technology may lead to increased motivation and engagement, besides allowing a wide range of possible tasks/exercises to be implemented in rehabilitation programs. The present review aims to investigate the possible benefits and efficacy of VR-based rehabilitation in individuals with SCI.

**Methods:**

An electronically systematic search was performed in multiple databases (PubMed, BVS, Web of Science, Cochrane Central, and Scielo) up to May 2019. MESH terms and keywords were combined in a search strategy. Two reviewers independently selected the studies in accordance with eligibility criteria. The PEDro scale was used to score the methodological quality and risk of bias of the selected studies.

**Results:**

Twenty-five studies (including 482 participants, 47.6 ± 9.5 years, 73% male) were selected and discussed. Overall, the studies used VR devices in different rehabilitation protocols to improve motor function, driving skills, balance, aerobic function, and pain level, as well as psychological and motivational aspects. A large amount of heterogeneity was observed as to the study design, VR protocols, and outcome measures used. Only seven studies (28%) had an excellent/good quality of evidence. However, substantial evidence for significant positive effects associated with VR therapy was found in most of the studies (88%), with no adverse events (88%) being reported.

**Conclusion:**

Although the current evidence is limited, the findings suggest that VR-based rehabilitation in subjects with SCI may lead to positive effects on aerobic function, balance, pain level, and motor function recovery besides improving psychological/motivational aspects. Further high-quality studies are needed to provide a guideline to clinical practice and to draw robust conclusions about the potential benefits of VR therapy for SCI patients. Protocol details are registered on PROSPERO (registration number: CRD42016052629).

## 1. Background

Spinal cord injury (SCI) is a common neurological condition that often results in long-term impairments in physical function and psychological and socioeconomic status [1]. Because of functional limitations in the sensory and motor systems [2], which may involve both lower and upper limb functions [3], SCI drastically affects independence and quality of life [4]. Different types of training and stimulation protocols are commonly used to induce or facilitate processes of neural regeneration and plasticity, which might lead to significant functional recovery after SCI [5]. Therefore, appropriate rehabilitation strategies are highly needed to regain sensorimotor function and reduce symptoms such as spasticity, imbalance, and neuropathic pain.

Recently, studies have used virtual reality (VR) as a promising tool for clinical rehabilitation in a variety of neurological disorders. For instance, VR-based technologies have been demonstrated to improve cognitive function after traumatic brain injury [6] and to promote balance control and gait recovery after stroke [7, 8], cerebral palsy [9], and SCI [10, 11]. VR makes use of advanced technologies (such as computers and multimedia peripherals) to provide an interactive and multidimensional simulated environment that users perceive as comparable with real-life experiences [11, 12]. The advantage of VR-based technologies over conventional rehabilitation therapies has been associated with increased motivation, engagement [13], and the wide range of possible tasks/exercises that might be implemented [10].

In fact, VR-based interventions in patients with SCI have been demonstrated to improve motor function [14–17], neuropathic pain [14, 18], balance [14, 19, 20], and aerobic function [21, 22]. Therefore, the use of VR-based rehabilitation in SCI clinical practice shows great promise; however, the evidence has not yet been formally reviewed or synthesized. To our knowledge, to date, there are no systematic reviews that integrate different results of the putative efficacy of VR in promoting sensorimotor recovery, as well as in reducing impairments and symptoms in patients after SCI. In order to provide a detailed and critical description of the effects of immersive or nonimmersive VR-based rehabilitation after SCI, we conducted a systematic review of published and unpublished studies up to May 2019.

## 2. Methods

This systematic review was performed in accordance with the Preferred Reporting Items for Systematic Reviews and Meta-Analyses (PRISMA) guidelines [23, 24] and registered as a predefined review protocol in PROSPERO (CRD42016052629).

### 2.1. Search Strategy

An initial search was performed electronically in PubMed, BVS, Web of Science, Cochrane Central, and Scielo databases in order to identify studies published between January 1, 1980, and May 1, 2019. The grey literature was also searched in ClinicalTrials.gov and Health Services Research projects, as well as in generic Internet research engines, to avoid missing relevant unpublished studies. The identified keywords and Medical Subject Headings (MeSH) were combined by boolean logic using the following terms: “virtual reality” OR “virtual reality immersion therapy” OR “virtual reality therapy” OR “reality therapy” OR “game(s)” AND “spinal cord injury(ies)” OR “spinal cord trauma” OR “paraplegia” OR “tetraplegia.” Additionally, the reference lists of all relevant literature were hand-searched to identify any additional suitable studies. Two reviewers performed the search independently.

### 2.2. Eligibility Criteria

Eligible studies included a sample with adults aged between 18 and 65 years (both genders) with traumatic or nontraumatic SCI who underwent immersive or nonimmersive VR-based rehabilitation. Only full scientific papers were included, regardless of the levels of lesion and the levels of disability (as assessed by American Spinal Injury Association (ASIA) Impairment Scale (AIS) classification) of the samples. Randomized controlled trials were included, along with nonrandomized controlled trials, quasiexperimental studies, and before and after studies. Studies reporting validity and/or development of VR games or devices as well as transversal comparisons designed to investigate physiological mechanisms rather than clinical efficacy assessments were excluded. Conference papers and abstracts, as well as papers written in languages other than English, Spanish, or Portuguese, were also excluded.

To increase confidence of the selection process, two reviewers (AA and JN) independently screened the title and abstract of each reference identified by the search strategy. The full-text article of all potentially relevant eligible references was subsequently retrieved and further examined independently. Discrepancies between reviewers were reconciled by discussion or a third independent reviewer (FM). All identified studies were stored in Mendeley®, and duplicates were removed.

### 2.3. Assessment of Risk of Bias

Two authors independently assessed the risk of bias and the methodological quality of the studies based on the PEDro scale, the most acceptable scale for rehabilitation research [25]. The scale assesses the presence or absence of randomization, allocation concealment, blinding of participants and/or researchers, homogeneity of the groups, intention-to-treat analysis, and presentation of statistical analysis. We considered the level of evidence as “excellent,” “good,” “fair,” or “poor” for PEDro scores in the ranges of 9-10, 6–8, 4-5, and <4, respectively [25].

### 2.4. Data Extraction

Two independent reviewers (AA and JN) filled out a data collection form on a customized Excel® spreadsheet. Data included information on participant characteristics (sample size, age, sex, cause of SCI, level of injury, type of SCI, ASIA impairment level, and time after injury), descriptive studies' characteristics (time of publication, continent/country, type of VR, and rehabilitation objectives), methodological details (study design, dropout rate, type of therapy, VR characteristics, number and time of sessions, frequency by week, follow-up time, and outcome measurements), VR effects (statistically significant or nonstatistically significant results), risk of bias, size effects, statistical power, and limitations. The results of extraction were compared, and divergences were resolved by consensus.

Study design was analyzed by considering the following aspects: randomization, blinding, presence of the control group, bias, internal and external validity, and statistical power. The statistical power was low when *β* − 1 < 80% (*α* = 0.05).

The participant characteristic data were grouped and expressed as mean or percentage for better visualization of the sample profile. To form the VR therapeutic guideline, we collected the following characteristics: type of VR (immersive or nonimmersive), type of the VR device (commercial or developed by authors), number and time of sessions, frequency of therapy by week, type of therapy (VR alone or combined with other interventions), motivational aspects, and adverse effects.

Finally, we observed the positive or negative effects of VR-based rehabilitation so as to perform considerations about the clinical practice based on statistical significance when *p* < 0.05.

## 3. Results

### 3.1. Search and Selection of Studies

**Figure 1:**
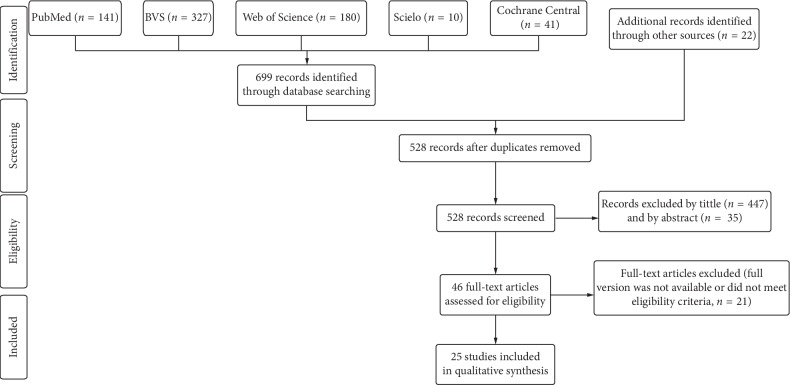
Flowchart of the search strategy of the published and unpublished literature and selection process (up to May 2019).

We identified 721 titles from searches in all databases (i.e., published and unpublished) and coming from the screened list of references (*n* = 2) for VR-based rehabilitation studies in individuals with SCI. We also found 20 unpublished studies, but none of them met the eligibility criteria because full text was not available. One hundred ninety-three studies were excluded by duplicates, 447 by title, and 35 by abstract. After full-text screening, 21 studies were excluded.  Figure 1 shows the selection process of studies identified and included, along with the reasons for exclusion. At the final stage, 25 studies were included in the qualitative analyses (Table 1).

### 3.2. Design of the Studies

From the 25 studies analyzed, 24 were prospective. Twelve of them used a pre-post design without control group. Thirteen were controlled in a parallel or crossover design (see Table 2). Only eleven studies used randomization to equally distribute the participants between groups of intervention. As to the blinding aspect, only two studies were double-blinded [10, 33], in which neither researchers nor participants knew how the sample was distributed within the groups. Three studies [18, 35, 39] were single-blinded, in which only researchers or participants knew group allocation (see Table 2). Another aspect that deserves attention is the sample size, which ranged from 6 to 54 participants. However, most of the studies had a small sample size and did not report statistical power associated with the observed effects.

The only nonprospective study was from Pozeg et al. [34], who used a 2-level factorial, randomized, repeated-measures design to investigate changes in perception of body ownership and neuropathic pain before and after experimental paradigms that combined virtual visual and tactile input.

### 3.3. VR Characteristics

The studies used either VR alone or VR paired with other therapy(ies). Fifteen applied treatment with only VR, while ten combined VR with occupational therapy and physiotherapy or conventional therapy. So, these studies show results about VR as an adjuvant treatment (see Table 2).

Despite different types of VR devices used in the studies (see Table 2), most of the protocols used the games to provide stimuli that encourage movements to improve motor function, balance, aerobic function, and pain. Some studies also used the walking control of an avatar in a virtual environment [17, 18], development of daily activities [10, 16], or training of driving skills [15, 29]. Both commercial and noncommercial VR devices were used in the studies.

The total number of VR rehabilitation sessions ranged from 1 to 36. The intervals between VR-based interventions also varied between studies, with a frequency ranging from 1 to 5 times a week (with sessions lasting from 1 to 90 minutes). However, some studies did not clearly report any of the following information: amount of sessions [15, 18, 21, 22, 26, 29, 39], duration [13, 15, 16, 20, 26, 29, 30], or frequency [15–18, 21, 26, 29] (see Table 2).

Finally, most of the studies reported results of the short-term effect of VR. Follow-up assessments to verify long-term effects were performed only in nine studies [13, 14, 16, 27, 31, 35–37, 39]. The follow-up time ranged between 4 and 30 weeks, and the pooled studies had a mean of 13.7 weeks of follow-up time (Table 2).

### 3.4. Outcome Measurements

Table 2 depicts all outcome measures used to assess the effects of the VR-based interventions. It is noteworthy that most of the studies used more than one instrument or scale. Moreover, many different scales were used to evaluate the motor function of lower or upper limbs, balance, and pain. Studies also quantified the independence and level of daily activities through the Spinal Cord Independence Measure Scale, Barthel Index, and Functional Independence Measure. On the other hand, all studies with quantification of the level of aerobic function in response to VR used similar measurements of the heart rate, oxygen consumption, and energy/metabolic expenditure. Five studies also used the quantitative variables such as the score or kinematic aspects of games or VR devices to evaluate the performance of SCI subjects in response to VR rehabilitation [15, 16, 28–30] (see Table 2).

Despite some studies have used the same outcome measurements, the variability in population characteristics, VR device aspects, and VR rehabilitation methods among the studies makes it very difficult and not relevant to perform a meta-analysis.

### 3.5. Participant Characteristics

The pooled sample of all studies included a total of 482 individuals with SCI. The dropout rate of participants was low, ranging from one to eight dropouts in nine studies. The majority of participants were men (73%), and the mean age was 47.6 ± 9.5 years.

The cervical level of injury was observed in 52% of the pooled sample, whereas 35% and 13% had thoracic and lumbar injuries, respectively. Incomplete lesions were more frequently observed (64.3% of the pooled sample). Regarding the ASIA impairment level, only 19 studies (*n* = 227 participants) presented complete data on this classification. In these studies, ASIA impairment level “A” was observed in 46% of the participants, whereas ASIA impairment levels “B,” “C,” and “D” were observed in 17%, 15%, and 22% of the participants, respectively. We highlight some studies did not present complete information about the cause of SCI (48%), level of injury (20%), type of SCI (16%), ASIA impairment level (24%), or time after injury (20%). In addition, two studies did not report most of the injury characteristics [20, 22].

The mean time after injury was 5 years (pooled analysis), which corresponds to the chronic stage of SCI. Only three studies included participants in the subacute stage of SCI (from ∼2 months to 1 year after injury) [29, 33, 35]. The detailed information and absolute numbers of each participant characteristic are shown in Table 3.

### 3.6. VR Effects

The main results concerning the effects of VR-based rehabilitation on individuals with SCI are summarized in Table 4. Overall, studies showed a statistically significant (*p* < 0.05) short-term improvement on motor function, aerobic performance, balance, pain, and psychological aspects. Only three studies reported effect sizes, which ranged from low to large treatment effects (Cohen's *d* values ranged from 0.41 to 1.95 and eta-squared values from 0.11 to 0.95) [33, 34, 39]. In addition, statistically significant long-term effects were observed on motor function [13, 14, 16, 31, 32, 35–37, 39], balance [14, 31, 35, 36], and pain [14, 34].

Interesting subjective results about positive VR motivational aspects such as better mood [14], high enjoyment [13, 14, 26], and improvements on satisfaction [27, 30] were reported in some studies.

### 3.7. VR Adverse Effects

Most of the studies did not directly report any adverse effect after VR therapy (88%). Only a reduced number of participants had a transient musculoskeletal pain (*n* = 2) [14, 17], physical fatigue (*n* = 4), and difficulties to maintain attention (*n* = 2) [17] because of the increased use of limbs during the sessions of therapy. Moreover, Carlozzi et al. [15] reported acute simulator sickness during the protocol in seven participants.

### 3.8. Risk of Bias

Risk of bias assessment showed that most of the analyzed studies involving the investigation of VR effects after SCI presented some level of potential bias. Only seven studies presented a low risk of bias associated with an excellent or good level of evidence (see Table 5).

### 3.9. Limitations of the Studies

All studies had design limitations (see Risk of Bias) and hence restriction on internal or external validity. Most of the studies did not perform randomization [13, 14, 16, 17, 19, 20, 22, 26, 29–32, 36, 37] or blinding [10, 13–17, 19, 22, 26, 28–32, 34, 36, 37].

The small and heterogeneous samples of SCI subjects with a wide range of injury levels, cause of injury, and time after injury were frequently observed in all studies. Therefore, the results of the studies included in this review cannot be generalized for the whole population with SCI, which represents an external validity limitation [40].

When accessible, the power of the statistical tests was low because of the small sample size used in most of the studies [41, 42]. In addition, only three studies included in this review reported effect sizes [33, 34, 39], an important estimator of clinical significance [43, 44].

Another limitation was the incomplete description of VR protocol characteristics (number of sessions, treatment frequency, duration, and training activities). Furthermore, a large variety of outcome measurements were reported among the studies, which preclude objective conclusions on specific aspects to be drawn.

## 4. Discussion

To the best of our knowledge, this systematic review is the first study aimed at investigating the effects of immersive or nonimmersive VR-based rehabilitation after SCI. Therefore, the present review provides a systematic overview and important guidelines for future research on VR-based rehabilitation for SCI individuals.

### 4.1. Summary of Main Results, Overall Completeness, and Applicability of Evidence

We included twenty-five studies involving a total sample of 482 subjects with SCI. The currently findings describe eighteen years of VR-based rehabilitation after SCI as an emerging research area. Based on the present results, reviewed studies applied VR therapy to (1) improve motor function or motor skills, (2) restore balance, (3) improve aerobic function, (4) reduce the pain level, or (5) provide better psychological/motivational aspects.

Seven of the studies presented an excellent or good level of evidence because they were controlled randomized clinical trials with satisfactory sample size [15, 18, 27, 28, 33, 35, 39] aimed to use immersive [15, 18, 27, 28, 33, 35] or nonimmersive VR [39] to improve upper limbs' motor function or reduce the pain level [18]. Six of these studies reported statistically significant positive effects of VR-based techniques associated with enhanced motor function [15, 28, 33, 35, 39] or reduction in pain levels [18].

Although high-quality evidence was limited, other statistically significant results were observed on aerobic function [21, 22, 26], balance [1, 13, 14, 19, 20, 31, 32, 37], and psychological aspects [7, 34, 37]. However, most of these studies presented important methodological limitations, and hence, the associated results should be interpreted with caution. Several studies also reported subjective positive results on motivational aspects of VR treatment [14, 27, 30, 39]. Indeed, previous studies have been considering VR as an interactive tool that provides a motivational environment associated with high engagement, which favors adherence to treatment [13, 45]. This is especially important when rehabilitation require repetitive movements or extensive protocols [46, 47].

Improved aerobic function and physical activity have been reported as beneficial effects of VR [21, 22, 26] on SCI. However, as few studies were aimed at assessing these aspects, the body of literature would certainly benefit from further investigations about the effects of VR-based protocols on aerobic performance, as VR protocols can be performed in safe and comfortable environments [48], besides allowing the trainers/therapists to set up the level of physical activity according to the physical fitness or functional limitation of the patient.

Most of the studies with adequate experimental design and positive effects on motor function investigated VR-based protocols paired with conventional rehabilitation [27, 28, 39]. Indeed, some ethical issues may arise regarding clinical trials designed to investigate isolated effects of a single (and sometimes novel) therapy. Such an investigation would require control groups to receive no treatment at all (even for conditions in which the current evidence points to effective treatments options), which might be considered not ethically appropriate [49]. Therefore, the present review suggests that VR might be an important adjunct tool for conventional therapy [50–54], considering benefits such as the wide variety of VR-based protocols that might be designed, the potential transferring of functional activities performed in the virtual environment to activities of daily living [55], the high adaptability of VR protocols according to the patient's limitations/preferences, and the positive effect of feedback during training on VR-based settings [10, 55, 56]. Additionally, some studies found positive effects of VR alone on motor function [13, 14, 17, 29, 31, 32, 36, 37], balance [13, 14, 19, 20, 31, 36, 37], aerobic function [21, 22, 26], and pain level [14, 34]. Thus, the present study cannot conclude whether VR-based rehabilitation is more effective than conventional therapy. Nevertheless, the positive effects reported provide the support to recommend the use of VR as an adjunct to conventional therapies in clinical practice.

Both commercial and noncommercial VR devices were used in the revised studies. The frequent use of noncommercial devices (i.e., customized and specifically built for the rehabilitation purposes at hand) is probably due to the requirement of contemplating the specific needs of the patients as a function of the level of their physical limitations after SCI [55]. Further studies are needed to determine whether there are different rehabilitation outcomes related to the use of specific types of VR devices.

Despite the large differences observed among the VR protocols used in the studies, both immersive and nonimmersive environments were able to induce the performance of a wide range of specific and global functional movements while promoting motivation to perform the activities [9, 50, 55, 57] and a safe rehabilitation setting with no adverse effects [9, 50, 55]. Taken together with the beneficial effects of the VR commented above, these aspects increase the potential use of VR as a rehabilitation tool after SCI.

### 4.2. Heterogeneity

The present review found a great level of heterogeneity, also reported in other VR systematic reviews in neurological disorders [9, 50, 54, 55]. Overall, the studies presented a wide range of VR characteristics and protocols. So, it remains unclear which device elements, VR type, number/frequency of sessions, and duration of VR-based rehabilitation are essential to induce optimal recovery after SCI. In addition, divergent outcome measurements were used in the studies reviewed. Similarly, heterogeneity in the injury level, lesion cause, injury type, and time after injury was commonly observed in the studies' samples. Indeed, SCI is a heterogeneous condition in nature, with nonlinear recovery [58, 59], which makes it difficult to run studies with homogeneous samples so as to establish the specific characteristics associated with better clinical outcomes. However, we observed that all studies applied VR-based rehabilitation in subjects with chronic SCI. So, the conclusion drawn in the present study can only be applied to individuals with chronic SCI, as none of the included studies assessed acute and subacute stages of SCI.

### 4.3. Quality of the Evidence

Despite the increased use of VR technology in neurorehabilitation study protocols [38, 54], it is not possible to draw strong conclusions about the efficacy of VR-based rehabilitation for patients with SCI because of the overall lack of methodological quality and statistical power observed in the current body of literature. Unfortunately, only seven studies included in the present systematic review had an excellent or good level of evidence (low risk of bias) (see Table 5). The same issue has been observed in other systematic reviews involving VR [9, 50, 54, 55].

The lack of adequate study design (randomized, controlled, and blinded studies), powered sample size, and absence of effect size report are the most important limitations of the studies reviewed here. Putative flaws in study design are associated with increased risk for selection, performance, or detection bias, thereby compromising internal validity [42, 60, 61]. Similarly, the lack of a control group in pre-post designs may have compromised the evidence of the treatment effect [62] and does not allow conclusions to be drawn about the nature of the observed effects [56]. In addition, studies with low statistical power and small samples might involve type II errors [41, 63] and hence low certainty of the detection of treatment effects.

Furthermore, some of the revised studies did not include information about the characteristics of VR rehabilitation protocols, reducing the possibility of replication by future studies. Future studies shall include appropriate description of training duration, frequency by week, and duration of sessions and detailed information about the virtual activities so as to allow putative associations between the observed effects and the specific training characteristics.

The limitations found in the revised studies preclude the detailed analysis of the effects of VR on SCI, as well as the grouping of results in a meta-analysis. Future studies should avoid methodological limitations and should use and report adequate statistic power so as to identify the effects of VR rehabilitation and ensure robustness for proper quantitative data analysis (meta-analysis).

### 4.4. Future Research

The present systematic review has important implications for future research. First, studies with participants in the acute and subacute stages of SCI are warranted. These phases are associated with greater potential for recovery and plasticity as compared to the chronic stage [2, 58] and hence might comprise an interesting scenario for VR-based rehabilitation. In this vein, future studies should be able to determine the VR applicability according to the injury level and comorbidities. Second, studies should explore the ideal VR characteristics related to success of rehabilitation and the effect duration (short-term and long-term effects). Third, studies should use the standardized outcome measurements. Fourth, statistical powered studies with adequate methods and design are warranted in order to reduce bias and provide reliable results. Finally, we highlighted the importance of the effect size report and detailed description of the VR protocol in future studies.

### 4.5. Potential Biases and Limitations in the Review Process

There are several limitations of this systematic review that must be pointed out: (1) although we conducted an extensive search in the published and unpublished literature, some relevant studies might not have been identified, (2) it is possible that publication biases exist in this field of research, (3) it was not possible to perform a meta-analysis because of heterogeneity of outcome measures and VR protocols, and (4) our findings are based on studies with a wide variety of methodological qualities and should therefore be interpreted with caution in terms of generalizability.

### 4.6. Agreements and Disagreements with Other Studies or Reviews

To our knowledge, no systematic review addressing the effectiveness of VR-based rehabilitation in subjects with SCI has been performed. However, we identified some current reviews on other neurologic disorders, such as stroke [50, 51, 54, 55], Parkinson's disease [55], and cerebral palsy [9].

Overall, these reviews reported positive effects of VR therapy on gait [50, 51, 54, 55], balance [50, 54], and motor function [9, 47, 52, 54]. The systematic review by Malloy and Milling [64] suggests beneficial effects of VR to reduce the pain level in a variety of pathological conditions. Therefore, VR emerges as a promising tool to improve the performance of daily activities and quality of life [55, 65].

Some of the studies report positive results when VR is used as an adjuvant therapy (i.e., VR paired with conventional therapy) [47, 50–52, 54], whereas other studies suggest that VR and conventional therapy may have similar effects [9, 55]. With the present review of the literature, it is not possible to conclude whether VR-based therapy in association with conventional therapy might provide more significant benefits for patients with SCI.

## 5. Conclusion

Overall, the studies that were included in the present systematic review reported a beneficial effect of VR therapy alone or VR associated with conventional rehabilitation. Initial evidence of VR to improve motor function, motor skills, balance, and aerobic function and to reduce the pain level was observed. Thus, our findings provide important implications for the VR-based rehabilitation research field. However, further studies should explore VR effects on SCI subjects considering the injury stage, level of lesion, and comorbidities. In addition, the related effects in response to VR characteristics and their specific applications should be studied. Similarly, evidence is required to provide information about VR long-term effects. Finally, high-quality studies are needed to provide robust guidelines and to draw conclusions about the potential benefits of VR before its integration into rehabilitation protocols for subjects with SCI.

## Figures and Tables

**Table 1:** General descriptive characteristics of included studies (*n* = 25).

*Time of publication*	
2000–2007	O'connor et al. [26]
2008–2012	Chen et al. [7]; Sayenko et al. [19]; Kowalczewski et al. [39]; Gil-Agudo et al. [28]; Sung et al. [29]
2013–2018	Villiger et al. [14]; Carlozzi et al. [15]; Gaffurini et al. [21]; Hasnan et al. [22]; Dimbwadyo-Terrer et al. [16]; D'Addio et al. [20]; Dimbwadyo-Terrer et al. [10]; Fizzotti et al. [30]; Villiger et al. [31]; Wall et al. [13]; Dimbwadyo-Terrer et al. [27]; Jordan et al. [18]; Roosink et al. [17]; An and Park [32]; Khurana et al. [33]; Pozeg et al. [34]; Prasad et al. [35]; van Dijsseldonk et al. [36]; Villiger et al. [37]

*Countries*	
Switzerland	Villiger et al. [14]; Villiger et al. [31]; Pozeg et al. [34]; Villiger et al. [37]
USA	Carlozzi et al. [15]; O'connor et al. [26]; Wall et al. [13]
Spain	Dimbwadyo-Terrer et al. [10]; Dimbwadyo-Terrer et al. [27]; Gil-Agudo et al. [28]; Jordan et al. [18]; Dimbwadyo-Terrer et al. [16]
Italy	Fizzotti et al. [30]; Gaffurini et al. [21]; D'Addio et al. [20]
Canada	Kowalczewski et al. [39]; Roosink et al. [17]
Japan	Sayenko et al. [19]
Sydney	Hasnan et al. [22]
Taiwan	Chen et al. [7]; Sung et al. [29]
Korea	An and Park [32]
India	Khurana et al. [33]; Prasad et al. [35]
Netherlands	van Dijsseldonk et al. [36]

*Type of VR*	
Immersive	Chen et al. [7]; Sayenko et al. [19]; Gil-Agudo et al. [28]; Carlozzi et al. [15]; Dimbwadyo-Terrer et al. [16]; Gaffurini et al. [21]; Hasnan et al. [22]; Villiger et al. [14]; D'Addio et al. [20]; Dimbwadyo-Terrer et al. [10]; Wall et al. [13]; Dimbwadyo-Terrer et al. [27]; Jordan et al. [18]; Roosink et al. [17]; Khurana et al. [33]; Pozeg et al. [34]; Prasad et al. [35]; van Dijsseldonk et al. [36]; Villiger et al. [37]
Nonimmersive	O'connor et al. [26]; Sung et al. [29]; Fizzotti et al. [30]; Villiger et al. [31]; Kowalczewski et al. [39]
Semi-immersive	An and Park [32]

*Objective of rehabilitation (domain)*	
Motor function	Kowalczewski et al. [39]; Gil-Agudo et al. [21]; Sung et al. [29]; Carlozzi et al. [15]; Dimbwadyo-Terrer et al. [16]; Villiger et al. [14]; Zimmerli et al. [38]; Dimbwadyo-Terrer et al. [10]; Fizzotti et al. [30]; Villiger et al. [31]; Wall et al. [13]; Dimbwadyo-Terrer et al. [27]; Roosink et al. [17]; An and Park [32]; Khurana et al. [33]; Prasad et al. [35]; Villiger et al. [37]; van Dijsseldonk et al. [36]
Aerobic function	O'connor et al. [26]; Gaffurini et al. [21]; Hasnan et al. [22]
Pain	Villiger et al. [14]; Jordan et al. [18]; Roosink et al. [17]; Pozeg et al. [34]
Balance	Sayenko et al. [19]; D'Addio et al. [20]; Wall et al. [13]; An and Park [32]; van Dijsseldonk et al. [36]; Villiger et al. [31]
Psychologic aspects	Chen et al. [7]; Pozeg et al. [34]; Villiger et al. [37]

**Table 2:** Methodological characteristics of included studies (*n* = 25).

Studies	Design	Sample/dropout (*n*/*n*)	Type of therapy	VR characteristics	*N*/*T* of sessions	Sessions per week	Follow-up time (weeks)	Outcome measurements
Villiger et al. [14]	Prospective, before and after design, noncontrolled, nonrandomized, nonblinded	14/0	VR	VR-augmented therapy system (games)	16 or 20/45 min	4 or 5	12–16	Numeric Rating Scale, 10-Meter Walk Test, lower extremity motor score, Spinal Cord Independence Measure, Walking Index for Spinal Cord Injury II, Patients' Global Impression of Change, Berg Balance Scale
Carlozzi et al. [15]	Prospective, controlled, randomized, nonblinded	54/2	VR	Virtual reality driving simulator	—	—	—	Simulator Sickness Questionnaire and software driving simulator variables
Dimbwadyo-Terrer et al. [10]	Prospective, controlled, randomized, nonblinded	15/6	VR + occupational therapy and physiotherapy	CyberGlove® + 3D objects (reach and release)	10/30 min	2	—	Muscle Balance, Barthel Index scale for functional capacity, Spinal Cord Independence Measure, Nine-Hole Peg Test, Jebsen–Taylor Hand Function
Dimbwadyo-Terrer et al. [27]	Prospective, controlled, double-blinded, randomized	31/0	VR + occupational therapy and physiotherapy	VR system Toyra (games)	15/30 min	3	12	Functional Independence Measure, Spinal Cord Injury Independence Measure, Motricity Index, Manual Muscle Test, Quebec User Evaluation of Satisfaction 2.0
Fizzotti et al. [30]	Prospective, before and after design, noncontrolled, nonrandomized, nonblinded	15/0	VR + traditional neurologic exercises	Apple iPad 2 (games)	6–36^*∗*^/—	2 or 3	—	Scores in the games and Trunk Recovery Scale
Gaffurini et al. [21]	Prospective, before and after design, noncontrolled, nonrandomized, nonblinded	10/0	VR	Wii Sports (games)	—/10 min	—	—	Oxygen consumption, pulmonary ventilation, heart rate, energy expenditure
Gil-Agudo et al. [21]	Prospective, controlled, randomized, nonblinded	10/0	VR + occupational therapy	Toyra system (games)	15/30 min	3	—	Variables of Toyra, Spinal Cord Injury Independence Measure, Nine-Hole Peg Test, Jebsen–Taylor Hand Function Test, Manual Muscle Test
Jordan et al. [18]	Prospective, controlled, single-blinded, randomized	35/0	VR	Visual illusory walking	—/20 min	—	—	Numeric Rating Scale and Quantitative Sensory Test
O'connor et al. [26]	Prospective, before and after design, noncontrolled, nonrandomized	10/0	VR	GAME^Wheels^ (games)	<3/3–12 min	—	—	Submaximal oxygen consumption, heart rate.
Roosink et al. [17]	Prospective, before and after design, noncontrolled, nonrandomized, nonblinded	9/0	VR	Visual illusory walking	2/90 min	NA	—	Motor imagery vividness, effort and speed, Basic Pain Data Set, Kinesthetic and Visual Imagery Questionnaire
Sayenko et al. [19]	Prospective, before and after design, noncontrolled, nonrandomized, nonblinded	6/0	VR	Game-based exercises	12/5 min	3	—	Force plate analysis system “Stabilan-01”
Villiger et al. [31]	Prospective, controlled, nonrandomized, nonblinded	9/0	VR	VR games	16 or 20/45 min	4 or 5	12–16	Longitudinal magnetic resonance
Wall et al. [13]	Prospective, before and after design, noncontrolled, nonrandomized	6/1	VR	Nintendo™ Wii Fit (games)	14/—	2	4	Timed Up and Go Test, 10-Meter Walk Test, 6-Minute Walk Test, Walking Index for Spinal Cord Injury II, Berg Balance Scale, Forward Functional Reach Test, Lateral Functional Reach Test, RAND SF-36
Hasnan et al. [22]	Prospective, before and after design, noncontrolled, nonrandomized, nonblinded	8/0	VR	Taxi Magic VR Trainer	—/32 or 48 min	2 or 3	—	Cardiorespiratory responses and power output
D'Addio et al. [20]	Prospective, controlled, randomized, nonblinded	30/0	VR + traditional physical therapy	Nintendo™ Wii Fit	36/—	3	—	Posturography (center of pressure data), Berg Balance Scale, Spinal Cord Independence Measure
Sung et al. [29]	Prospective, before and after design, noncontrolled, nonrandomized, nonblinded	12/0	VR	Driving simulator	—	—	—	Simulator performance measure
Dimbwadyo-Terrer et al. [16]	Prospective, controlled, nonrandomized, nonblinded	20/2	VR + occupational therapy and physiotherapy	Toyra system	12/—	4	12	Kinematic variables, Motor Index, Muscle Balance, Functional Independence Measure, Spinal Cord Independence Measure II, Barthel Index
Kowalczewski et al. [39]	Prospective, controlled, single-blinded, randomized	21/8	VR + conventional exercise therapy	ReJoyce Workstation	—/60 min	—	30	Action Research Arm Test and ReJoyce Automated Hand Function Test
Chen et al. [7]	Prospective, controlled, nonblinded, randomized	30/0	VR	EON Studio 4.0	—/—	—	—	Endurance, Borg's Rating-of-Perceived-Exertion Scale, Activation-Deactivation Adjective Check List, Simulator Sickness Questionnaire
An and Park [32]	Prospective, before and after design, noncontrolled, nonrandomized, nonblinded	10/0	VR	Interactive Rehabilitation and Exercise (IREX; GestureTek, Toronto, Canada)	18/30 min	3	—	Limit of stability, Berg Balance Scale, Timed Up and Go Test, Activities-Specific Balance Confidence Scale, Walking Index for Spinal Cord Injury II
Khurana et al. [33]	Prospective, before and after design, controlled, randomized, double-blinded	36/6	VR + conventional physiotherapy	Sony PlayStation 2 and EyeToy (Sony Computer Entertainment Inc., Beijing, China)	20/45 min	4	—	Modified Functional Reach Test, t-shirt test, self-care components of the Spinal Cord Independence Measure III
Pozeg et al. [34]	Single-session, cross-sectional, controlled, randomized, nonblinded	40/0	VR + tactile stimulation (synchronous and asynchronous)	Virtual leg illusion and full-body illusion	1/1 min	1	—	Sense of leg ownership (questionnaires) and perceived neuropathic pain (visual analogue scale pain ratings)
Prasad et al. [35]	Prospective, pilot, controlled, single-blinded, randomized	22/2	VR + conventional hand therapy and strength training	Wii Sports Resort game (Table Tennis, Swordplay Speed Slice, Bowling, and Cycling)	12/60 min	3	6	Capabilities of Upper Extremity Questionnaire, Box and Block Test for gross motor dexterity, Spinal Cord Independence Measure-Self Report, World Health Organization Quality of Life-BREF
van Dijsseldonk et al. [36]	Prospective, before and after design, noncontrolled, nonrandomized, nonblinded	17/2	VR	Gait Real-time Analysis Interactive Lab (GRAIL) training	12/60 min	2	20	Gait (spatiotemporal parameters and stability measures) and Activities-specific Balance Confidence (ABC) Scale
Villiger et al. [37]	Prospective, before and after design, noncontrolled, nonrandomized, nonblinded	12/1	VR	Mobile prototype of the YouKicker system (YouRehab AG, Schlieren, Switzerland) for the lower limbs	16–20/30–45 min	4-5	10–13	Muscle strength, balance, and mobility: lower extremity motor score, Berg Balance Scale, Timed Up and Go Test, 10-Meter Walk Test, 6-Minute Walk Test, Spinal Cord Independence Measure III, Walking Index for Spinal Cord Injury II, Motivational Scale and Global Impression of Change

*Note. n* or *N*, number; VR, virtual reality; *T*, time; NA, not applicable; —, information not available. ^*∗*^The number is not specified (ranges from min. to max.).

**Table 3:** Characteristics of patients with SCI included in the individual studies (*n* = 25).

Studies	Sample (*n*)	Age (years) (mean)	Sex		Cause of SCI		Level of SCI			Type of injury		AIS				Time after injury (years) (mean)
			F	M	T	NT	C	T	L	CO	IN	A	B	C	D	
Villiger et al. [14]	14	52.7	5	9	7	8	7	7	0	0	14	0	0	2	12	5.5
Carlozzi et al. [15]	52	37.9	7	45	42	10	—	—	—	—	—	—	—	—	—	8.9
Dimbwadyo-Terrer et al. [10]	9	49.5	2	7	6	3	1	8	0	8	1	8	0	0	1	5.41
Dimbwadyo-Terrer et al. [27]	31	37.4	9	22	29	2	31	0	0	21	10	21	10	0	0	4.9
Fizzotti et al. [30]	15	37	12	3	—	—	—	—	—	12	3	12	2	1	0	—
Gaffurini et al. [21]	10	40	0	10	—	—	2	8	0	10	0	10	0	0	0	—
Gil-Agudo et al. [21]	10	42.6	6	4	6	4	10	0	0	5	5	5	5	0	0	5
Jordan et al. [18]	15	47.5	2	6	7	1	4	4	0	5	3	5	2	1	0	16.1
O'connor et al. [26]	10	41.9	3	7	—	—	0	10	0	—	—	—	—	—	—	13.8
Roosink et al. [17]	9	53	2	7	9	0	3	5	1	6	3	6	1	2	0	6.7
Sayenko et al. [19]	6	41	1	5	—	—	2	4	0	0	6	0	0	4	2	9.16
Villiger et al. [31]	9	47.1	4	5	—	—	5	4	0	0	9	0	0	0	9	3.2
Wall et al. [13]	6	58.6	0	6	—	—	6	0	0	0	6	0	0	0	6	7.6
Hasnan et al. [22]	8	—	—	—	—	—	—	—	—	—	—	—	—	—	—	—
D'Addio et al. [20]	30	43	—	—	—	—	—	—	—	0	30	—	—	—	—	—
Sung et al. [29]	12	28.5	2	10	11	1	3	7	2	8	4	—	—	—	—	1.93
Dimbwadyo-Terrer et al. [16]	18	37.7	7	11	17	1	18	0	0	11	7	11	7	0	0	5.17
Kowalczewski et al. [39]	13	35.9	6	7	—	—	13	0	0	4	9	4	—	—	—	3.62
Chen et al. [7]	30	48.2	16	14	—	—	0	0	30	0	30	—	—	—	—	—
An and Park [32]	10	44.2	4	6	—	—	8	2	0	0	10	0	0	4	6	19.2
Khurana et al. [33]	30	29.6	2	28	30	0	0	30	0	—	—	—^*∗*^	—	0	0	∼0.25
Pozeg et al. [34]	20	47.3	2	18	18	2	0	—	—^*∗∗*^	15	5	15	3	2	0	17.1
Prasad et al. [35]	20	28.3	1	21^#^	20	0	20	0	0	5	17^#^	5	9	4	4^#^	1.07^#^
van Dijsseldonk et al. [36]	15	59	4	11	—	—	—	—	—	0	15	0	0	13	2	3.5
Villiger et al. [37]	11	60	—	—	7	4	5	5	1	0	11	0	0	1	10	7.6

*Note*. *n*, number; SCI, spinal cord injury; AIS, American Spinal Injury Association (ASIA) Impairment Scale; —, information not available. ^*∗*^Participants were classified as A or B in AIS. ^*∗∗*^Lesions ranged from high thoracic (T2) to lumbar (L2). ^#^Data prior to dropout of 2 participants.

**Table 4:** Synthesis of the VR short-term effects by domain (motor function, aerobic function, pain, balance, or psychologic aspects) of statistically significant or nonsignificant results of individual studies (*n* = 25).

Studies	Statistically significant results (*p* < 0.05)					Statistically nonsignificant results				
	Motor function	Aerobic function	Pain	Balance	Psychologic aspects	Motor function	Aerobic function	Pain	Balance	Psychologic aspects
Villiger et al. [14]	✓		✓	✓						
Carlozzi et al. [15]	✓									
Dimbwadyo-Terrer et al. [10]						✓				
Dimbwadyo-Terrer et al. [27]						✓				
Fizzotti et al. [30]	✓									
Gaffurini et al. [21]		✓								
Gil-Agudo et al. [28]	✓								✓	
Jordan et al. [18]			✓							
O'connor et al. [26]		✓								
Roosink et al. [17]	✓							✓		
Sayenko et al. [19]				✓						
Villiger et al. [31]	✓			✓						
Wall et al. [13]	✓			✓						
Hasnan et al. [22]		✓								
D'Addio et al. [20]				✓						
Sung et al. [29]	✓									
Dimbwadyo-Terrer et al. [16]	✓^*∗*^					✓			✓	
Kowalczewski et al. [39]	✓									
Chen et al. [7]					✓					
An and Park [32]	✓^*∗∗*^			✓					✓	
Khurana et al. [33]	✓^*∗∗∗*^					✓				
Pozeg et al. [34]			✓^#^		✓			✓		
Prasad et al. [35]	✓									
van Dijsseldonk et al. [36]	✓^##^			✓		✓				
Villiger et al. [37]	✓^###^			✓	✓	✓				

*Note*. ^*∗*^The study had statistically significant results only in one functional aspect measured. ^*∗∗*^Overall limits of stability significantly improved, but directional forward and backward limits of stability did not differ significantly after therapy. ^*∗∗∗*^Modified Functional Reach Test (mFRT) and self-care components of the Spinal Cord Independence Measure III (SCIM III) significantly improved, but t-shirt test did not differ significantly after therapy. ^#^Significant pain reduction when the lower back was stimulated synchronously with the virtual legs but no significant reductions for other conditions. ^##^Significant effects on 4 out of 9 spatiotemporal and stability measures of gait. ^###^Significant improvements on LEMS, BBS, and TUG, but no significant changes on 6minWT, SCIM III, and WISC-III.

**Table 5:** PEDro scale scores, assessment of the level of evidence, and risk of bias of individuals studies (*n* = 25).

Studies	PEDro scale score	Level of evidence	Risk of bias
Villiger et al. [14]	5	Fair	High
Carlozzi et al. [15]	6	Good	Low
Dimbwadyo-Terrer et al. [10]	5	Fair	High
Dimbwadyo-Terrer et al. [27]	10	Excellent	Low
Fizzotti et al. [30]	4	Fair	High
Gaffurini et al. [21]	5	Fair	High
Gil-Agudo et al. [28]	6	Good	Low
Jordan et al. [18]	7	Good	Low
O'connor et al. [26]	3	Poor	High
Roosink et al. [17]	5	Fair	High
Sayenko et al. [19]	4	Fair	High
Villiger et al. [31]	4	Fair	High
Wall et al. [13]	5	Fair	High
Hasnan et al. [22]	4	Fair	High
D'Addio et al. [20]	4	Fair	High
Sung et al. [29]	4	Fair	High
Dimbwadyo-Terrer et al. [16]	5	Fair	High
Kowalczewski et al. [39]	7	Good	Low
Chih-Hung et al. (2009)	5	Fair	High
An and Park [32]	4	Fair	High
Khurana et al. [33]	9	Excellent	Low
Pozeg et al. [34]	5	Fair	High
Prasad et al. [35]	6	Good	Low
van Dijsseldonk et al. [36]	4	Fair	High
Villiger et al. [37]	4	Fair	High
